# Global lncRNA expression signature in pre-metastatic lung and their regulatory effects in pulmonary metastasis

**DOI:** 10.3389/fimmu.2024.1506561

**Published:** 2024-11-29

**Authors:** Huifang Shi, Peng Wang, Jiaan Wang, Lei Chen, Yan Qin, Jie Lv

**Affiliations:** ^1^ Clinical Laboratory, The Rizhao People’s Hospital Affiliated to Jining Medical University, Rizhao, Shandong, China; ^2^ Clinical Laboratory, Rizhao Center for Disease Control and Prevention, Rizhao, Shandong, China; ^3^ Blood Transfusion Department, The Rizhao People’s Hospital Affiliated to Jining Medical University, Rizhao, Shandong, China

**Keywords:** pre-metastatic niche, lncRNAs, alveolar macrophage, M2 polarization, metastasis

## Abstract

**Background:**

Lung metastasis has garnered significant attention due to its prevalent occurrence. Pre-metastatic niche (PMN) establishment is a critical prerequisite for the onset of lung metastasis. Emerging evidence indicates that long noncoding RNAs (lncRNAs) play pivotal roles in the metastatic cascade to the lungs. However, the relationship between lncRNA expression profiles and the formation of PMN remains uncharacterized. This study aims to explore the expression profiles and potential roles of lncRNAs in the context of pre-metastatic lung microenvironment.

**Methods:**

RNA sequencing was utilized to elucidate the lncRNA landscape in pre-metastatic lung of murine models. Gene Ontology (GO) and Kyoto Encyclopedia of Genes and Genomes (KEGG) enrichment analyses were performed to infer the prospective functions of the differentially expressed lncRNAs. Among these, lncRNA Gm5144-202 in alveolar macrophages (AMs) was further scrutinized for its role in driving M2 macrophage polarization, facilitating the formation of PMN, and orchestrating the apoptosis, proliferation, and migration of tumor cells *in vitro*.

**Results:**

A total of 232 lncRNAs exhibited differential expression in pre-metastatic murine lungs compared to normal controls, predominantly enriching pathways such as PI3K-Akt signaling, calcium signaling, neuroactive ligand-receptor interaction, and NF-κB signaling. Notably, lncRNA Gm5144-202 exhibited the most pronounced difference, with elevated level in alveolar macrophages (AMs) during the pre-metastatic phase. Silencing of lncRNA Gm5144-202 impeded the polarization of M2-like macrophages, suppressed the expression of factors critical for the formation of the PMN, and inhibited tumor cell invasion.

**Conclusions:**

Our research delineated the lncRNA expression profiles in pre-metastatic pulmonary tissues and identified, for the first time, the pivotal role of lncRNA Gm5144-202 in modulating M2 macrophage polarization and tumor cell invasiveness. Consequently, targeting lncRNA Gm5144-202 holds substantial promise for translational applications aimed at mitigating pulmonary metastasis.

## 1 Introduction

Metastasis accounts for the majority of mortality associated with cancer (1). Pulmonary metastasis has received much attention due to its high prevalence in malignant diseases. Approximately 50% of patients who succumb to malignancies are found to have lung metastases post-mortem (2). Various cancer types, including melanoma, breast cancer, hepatic carcinoma, and colorectal cancer, exhibit a predilection for lung colonization (3). The interplay between cancer cells and the tumor microenvironment dictates the metastatic trajectory. The introduction of the “pre-metastatic niche” (PMN) concept has expanded our understanding of organotropic metastasis mechanisms. PMN is defined as a conducive microenvironment that facilitates the colonization of circulating tumor cells, thereby enhancing metastatic progression (1). Numerous cellular and molecular constituents have been recognized for their role in PMN establishment, with these mediators being synthesized not only by primary tumor cells but also by resident myeloid and stromal cells. Consequently, pre-metastatic lung niche establishes a favorable microenvironment for lung metastasis seeding. Nevertheless, it is imperative to conduct comprehensive investigations into the functions of these myeloid and stromal cell populations, as well as their associated molecular factors, in the formation of PMN within the metastatic organ.

Noncoding RNAs (ncRNAs), devoid of protein-coding capability, are pivotal in modulating distant tumor metastasis. NcRNAs encompass small interfering RNA (siRNAs), microRNAs (miRNAs), circular RNAs (circRNAs), long non-coding RNAs (lncRNAs), among others, categorized based on their structure and length (4). Nucleic acid drugs have exhibited huge potential in disease prevention and treatment in view of their powerful molecular targeted therapeutic properties (5, 6). For example, STP705, comprised of two siRNA oligonucleotides directly targeting TGFβ1 and COX2, has been evaluated in at least four phase I and/or II trials, and exhibited considerable clinical potential for treatment of carcinoma (7). Recent studies have also underscored the significant role of miRNAs in PMN formation, implicating their involvement in signaling pathways and gene expression linked to inflammation, immunosuppression, angiogenesis, and PMN reprogramming (8). However, the functional understanding of other ncRNA types, such as lncRNAs, in PMNs remains limited. LncRNAs, defined as ncRNAs exceeding 200 nucleotides without protein-coding potential, exhibit low evolutionary sequence conservation and are prevalent in the eukaryotic transcriptome (9). Emerging evidence highlights their crucial roles in gene expression modulation (10), chromatin dynamics (11), posttranslational regulation (12), and alternative splicing (13). Numerous studies have confirmed that lncRNAs are implicated in various diseases, such as autoimmune diseases (14), cancer (15), cardiovascular diseases (16) and infectious diseases (17). Cancer metastasis demonstrates organ-specific tropism, with certain lncRNAs identified as key regulators influencing the preferential metastatic sites (18). Additionally, some lncRNAs have emerged as biomarkers to identify tumors with increased metastatic potential and may inspire innovative therapeutic approaches, with numerous lncRNAs have demonstrated promise as novel candidates for antisense oligonucleotide therapy in preclinical animal studies. Nevertheless, whether there are lncRNAs that mediating lung metastasis, and their potential functions for PMN reconstruction have yet to be clarified.

This study initially identified lncRNA different expression profiles in lungs from mice with or without pre-metastasis. Subsequent bioinformatic analyses were performed to predict potential biological processes and signaling pathway interaction networks. Notably, the candidate lncRNA Gm5144-202 exhibited the most significant differential expression and was implicated in tumor cell migration and pro-tumor macrophage polarization. Given the extensive and critical roles of lncRNAs in pre-metastatic lungs, a comprehensive understanding of their regulatory mechanisms could unveil novel diagnostic and therapeutic targets for preventing and managing lung metastasis.

## 2 Materials and methods

### 2.1 Mice and sample collection

Female C57BL/6J mice, aged 8 to 10 weeks, were subcutaneously administered with 1×10^6^ B16F10 melanoma cells to construct a pulmonary pre-metastatic niche model, as previously described (19). After two weeks, the mice’s lungs were harvested post-perfusion with pre-chilled PBS to eliminate peripheral blood cells. The excised lung tissues were rapidly frozen in liquid nitrogen and stored at −80°C until further analysis. All mice utilized in these experiments were sourced from the Animal Experimental Center of Shandong Province (Jinan, China) and housed under specific pathogen-free (SPF) conditions. The study received approval from the Animal Care Committee of Jining Medical College (Approval No. JNMC-2024-DW-069).

### 2.2 Cell culture

The B16F10 melanoma cell line and the immortalized murine alveolar macrophage cell line MH-S were obtained from the American Type Culture Collection (ATCC). The cells were cultured in Dulbecco’s Modified Eagle’s Medium (DMEM; GIBCO, NY, USA) or RPMI 1640 Medium (GIBCO, NY, USA) supplemented with 10% fetal bovine serum (FBS; HyClone, Logan, UT, USA). All cell cultures were maintained at 37°C in a 5% CO_2_ humidified incubator. To prepare B16F10-conditioned medium (TCM) for the MH-S coculture assay, B16F10 cells at 80% confluence were rinsed twice with PBS and cultured in RPMI 1640 Medium for 24 hours.

### 2.3 Total RNA extraction

Total RNA was extracted from fresh-frozen lung tissues and cells using TRIzol reagent (Invitrogen, USA) following manufacturer’s protocol. Then the purity and concentration of isolated RNA were assessed via a NanoDrop 2000 instrument. RNA integrity was analyzed using the Agilent 2100/LabChip GX system (Agilent, Germany).

### 2.4 Transcriptome high throughput sequencing and bioinformatical analysis

Following RNA quality validation, library construction was performed as follows: ribosomal RNA (rRNA) was depleted using specific rRNA probes, and the remaining RNA was fragmented randomly. cDNA synthesis and purification were conducted according to the kit protocol. The resulting library was assessed using the Qubit 3.0 fluorometer and quantitative PCR (Q-PCR), and sequencing was conducted on the Illumina NovaSeq 6000. Bioinformatics analysis was performed via BMKCloud (www.biocloud.net). Briefly, clean reads were obtained through filtering out low-quality reads, adapters, as well as poly-N reads from raw data, which were then aligned to the reference genome. Differential expression and target gene prediction of lncRNAs, along with their potential functions, were subsequently conducted. The sequencing data has been uploaded in the GEO repository (GSE274656).

### 2.5 RT-qPCR

Reverse-transcription of extracted RNA was performed to get cDNAs according to the manufacturer’s guidelines (Thermo Fisher Scientific, USA). SYBR Green Master Mix (TaKaRa, China) was utilized for cDNA amplification on an ABI 7500 system (Thermo Fisher Scientific, USA). Primer sequences are listed in **Table 1**, with primer specificity verified by melting curves. The expression level of genes was normalized to β-actin and calculated using 2^-ΔΔCt^.

**Table 1:** Primers used for RT-qPCR.

Genes	Primer sequence (5’-3’)
lncRNA 5330413P13Rik-201	Forward: TCCTGTTTCAGGCAGCAGACAAC
lncRNA 5330413P13Rik-201	Reverse: TGTGCCATCTGTGCGTGTTGAGA
lncRNA BC106179-202	Forward: CCTGTCTTTCAGATTGTCTTCCTCCAT
lncRNA BC106179-202	Reverse: GTCCTCGCTCCTGTTTCCATCTT
lnc 9330198N18Rik-201	Forward: GCGTCTTCTAAGCCCTCTGTCCT
lnc 9330198N18Rik-201	Reverse: CAGACGGTGGGGAGTGGCTATTT
lncRNA Gm20634-201	Forward: AGCCACCCACTGAATCAGATAAAGA
lncRNA Gm20634-201	Reverse: AACCAAAGAGCGACAACAAACTACC
lncRNA E230016M11Rik-202	Forward: AACGCACGGATGAGGGTGAAATG
lncRNA E230016M11Rik-202	Reverse: TGGAGGGAAGCAGGTGGGTAGAG
lncRNA Gm5144-202	Forward: GCGAAACTTGTTGCTTCCGATGT
lncRNA Gm5144-202	Reverse: CTCCGTCTGCATCGCTGTGGTAG
Arg1	Forward: GACAGGGCTCCTTTCAGGAC
Arg1	Reverse: CTTGGGAGGAGAAGGCGTTT
TGFβ	Forward: GAAGCGGACTACTATGCTAAAGAGG
TGFβ	Reverse: GGTAACGCCAGGAATTGTTGCTAT
VEGFA	Forward: TGTAACGATGAAGCCCTGGAGTG
VEGFA	Reverse: CAAACAAATGCTTTCTCCGCTCT
S100A8	Forward: TGCCCTCTACAAGAATGACT
S100A8	Reverse: CTTGTGGCTGTCTTTGTGAG
MMP9	Forward: CTTCTGGCGTGTGAGTTTCCA
MMP9	Reverse: ACTGCACGGTTGAAGCAAAGA
Bv8	Forward: GCATGACAGGAGTCATCATTTT
Bv8	Reverse: AAATGGCAGGATATCAGGAAA
CXCL1	Forward: ATGGCTGGGATTCACCTCAA
CXCL1	Reverse: CAAGGGAGCTTCAGGGTCAA
CXCL2	Forward: GCCCAGACAGAAGTCATAGCC
CXCL2	Reverse: TCAGTTAGCCTTGCCTTTGTTC
β-actin	Forward: AACAGTCCGCCTAGAAGCAC
β-actin	Reverse: CGTTGACATCCGTAAAGACC

### 2.6 CCK8 assay

CCK8 assay was carried out to assess the proliferation of B16F10 cells. Briefly, B16F10 cells cocultured with medium containing 30% supernatant from MH-S cells in various experimental conditions were plated into 96-well plates. Next, 10 μl CCK8 (Beyotime Inc., China) was supplemented into each well and incubated for one hour in the dark at 37°C. The absorbance at 450 nm was then measured with a microplate reader (BIO-RAD iMark, USA).

### 2.7 Cell apoptosis analysis

The Annexin V-FITC apoptosis detection kit (Beyotime Inc., China) was utilized to analyze cell apoptosis by flow cytometry. In brief, B16F10 cells were grown in 30% conditioned medium from MH-S cells in different groups. The B16F10 cells were then harvested and rinsed with PBS. Subsequently, 195 μl of Annexin V-FITC binding buffer was added to gently resuspend the cells, followed by the addition of 5 μl Annexin V-FITC and 10 μl PI. After a 10-minute incubation at room temperature, flow cytometry analysis was promptly performed.

### 2.8 Transwell assay

For transwell assays, 1×10^5^ B16F10 tumor cells were seeded in the upper chamber (8 µm pore size, Corning, USA) with 0.2 mL of DMEM medium without serum, while MH-S cells from different groups were placed in the lower chamber. After a 24-hour incubation, the remaining cells in the upper chamber were wiped off with a cotton swab, and cells that had penetrated to the lower surface of the membrane were stained with crystal violet ((Beyotime Inc., China) for 20 minutes.

### 2.9 Fluorescence *in situ* hybridization and fluorescence immunohistochemistry

The expression and localization of lncRNA Gm5144-202 in pre-metastatic lungs and MH-S cells were examined through FISH assay. FISH probe (GenePharma, China), tagged with 5’ biotin, was combined with Cy3 at a 4:1 ratio and incubated overnight at 37°C. For lncRNA Gm5144-202 detection, probe mixture was utilized, with the sequences were: 5’-CGTAAATTGCTGCGTGA-3’; 5’-GAGCGGAATCATTTATCCAACA-3’; 5’-AGCCACCAATTTGGAGAAACAT-3’. The negative control sequence was: 5’-TGCTTTGCACGGTAACGCCTGTTTT-3’. For dual staining of FISH and fluorescent immunohistochemistry, primary anti-mouse F4/80 antibody (BM8, Biolegend, USA) was applied and incubated at 4°C overnight, and then labeled with FITC-conjugated secondary antibody (ZSGB-BIO, China). Subsequently, DAPI (Beyotime Inc., China) was stained to display cell nucleus. The fluorescent images were captured on a 3DHISTECH microscope (3DHISTECH Ltd., Hungary).

### 2.10 Transfection of siRNA duplexes

Small interfering RNAs (siRNAs) targeting lncRNA Gm5144-202 or non-specific control siRNAs were synthesized by GenePharma, with sequences provided in **Table 2**. SiRNA transfection to MH-S cells was conducted by using Lipofectamine 3000 (Invitrogen, USA) as per the instructions.

**Table 2:** siRNAs sequences used in this study.

Target genes	Sequence (5’-3’)
lncRNA Gm5144-202^#^1	Sense: GGGAAAUGUUUCUCCAAAUTT
lncRNA Gm5144-202^#^1	Antisence: AUUUGGAGAAACAUUUCCCTT
lncRNA Gm5144-202^#^2	Sense: AAACUUGUUGCUUCCGAUGTT
lncRNA Gm5144-202^#^2	Antisence: CAUCGGAAGCAACAAGUUUTT
lncRNA Gm5144-202^#^3	Sense: GGAUUGUGGAGAAGUCUCATT
lncRNA Gm5144-202^#^3	Antisence: UGAGACUUCUCCACAAUCCTT
lncRNA Gm5144-202^#^4	Sense: CACGCAGCAAAGACAUUUATT
lncRNA Gm5144-202^#^4	Antisence: UAAAUGUCUUUGCUGCGUGTT
Negative control	Sense: UUCUCCGAACGUGUCACGUTT
Negative control	Antisense: ACGUGACACGUUCGGAGAATT

### 2.11 Western blot analysis

Total protein from MH-S cells from different groups were extracted with RIPA lysis solution (Beyotime, China) according to the manufacturer’s instructions. The supernatants were collected and protein concentrations were determined by BCA assay (Beyotime, China). The protein samples were subjected with SDS-PAGE and subsequently transferred to the polyvinylidene fluoride (PVDF) membrane. After blocking, the membrane was incubated overnight at 4°C with the following primary antibodies: anti-Akt (1:2000, #4691S, Cell Signaling Tech, USA), anti-phospho-Akt (1:3000, 66444-1-Ig, proteintech, China), anti-NF-κB p65 (1:2000, WL01273b, Wanleibio, China), anti-phospho-NF-κB p65 (1:2000, GB11142-1, Servicebio, China), and anti-GAPDH (1:2000, TA-08, ZSGB-BIO, China). Secondary antibodies used were goat anti-rabbit or goat anti-mouse IgG-HRP (ZSGB-BIO, China).

### 2.12 Statistical analysis

Statistical analyses were conducted via GraphPad Prism 5 software. The Student’s t-test or a two-way analysis of variance (ANOVA) was applied for comparisons between groups, and difference with p value less than 0.05 was considered statistically significant.

## 3 Results

### 3.1 Global analysis of lncRNA expression profiles in normal and pre-metastatic lungs

**Figure 1:**
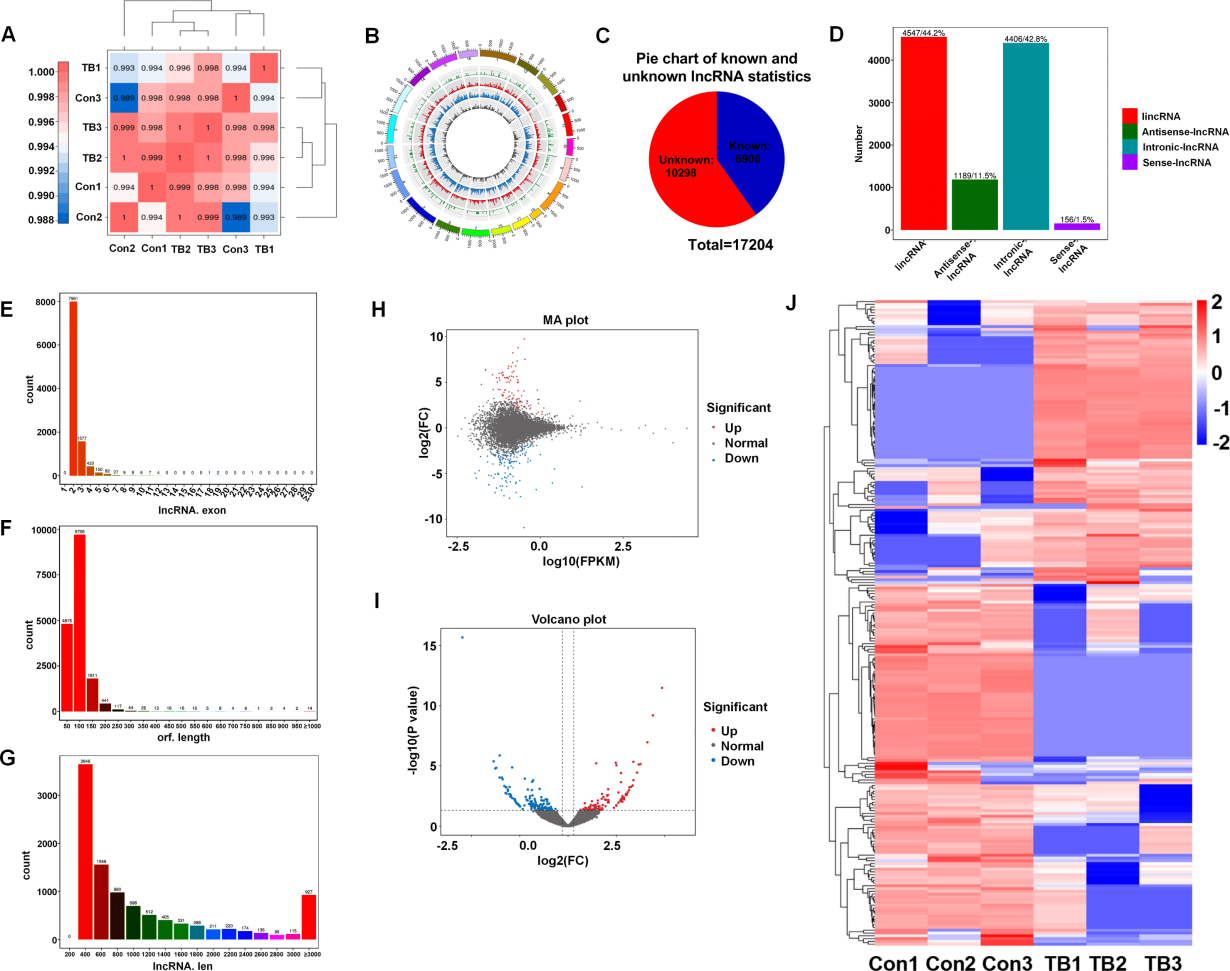
Overview of lncRNA characteristics in murine normal and pre-metastatic lungs. **(A)** Heatmap illustrating sample correlation. **(B)** Circos plot displaying chromosomal distribution of identified lncRNAs, categorized into genomic chromosomes, sense lncRNA, intergenic lncRNA (lincRNA), antisense lncRNA, and intronic lncRNA, from outer to inner circles. **(C)** Pie chart depicting the number of known versus novel lncRNAs identified. **(D)** Quantitative and percentage representation of lincRNA, antisense lncRNA, intronic lncRNA, and sense lncRNA among novel lncRNAs. **(E)** Distribution of exon numbers among novel lncRNAs. **(F)** Distribution of ORF lengths in novel lncRNAs. **(G)** Length distribution of novel lncRNAs. **(H)** MA plot showcasing differentially expressed lncRNAs between pre-metastatic and normal lung groups. **(I)** Volcano plot of differentially expressed lncRNAs between pre-metastatic and normal lung groups. **(J)** Heatmap depicting expression profiles of differentially expressed lncRNAs in normal and pre-metastatic lungs.

Lungs from normal mice and B16F10-bearing mice (2 weeks post-inoculation) were collected for transcriptome sequencing as previously described to delineate the lncRNA expression profiles in pre-metastatic lungs (20). Approximately 100 million reads were obtained for each sample, with unique mapped reads exceeding 90% for all samples (**Table 3**). Biological replicate correlation was evaluated using Pearson’s correlation coefficient, yielding r^2^ values more than 0.9, indicative of strong sample correlation (**Figure 1A**). The chromosomal distribution of identified lncRNAs was illustrated in a circos diagram, with genomic chromosomes on the outermost ring, followed by sense lncRNA, intergenic lncRNA (lincRNA), antisense lncRNA, and intronic lncRNA loops (**Figure 1B**). A total number of 17,204 lncRNAs were characterized, encompassing 6,906 known lncRNAs and 10,298 novel lncRNAs (**Figure 1C**). Among the novel lncRNAs, lincRNAs and intronic lncRNAs were predominant, accounting for 44.2% (4,547/10,298) and 42.8% (4,406/10,298) respectively (**Figure 1D**). Most novel lncRNAs consisted of two exons (**Figure 1E**), with open reading frame (ORF) lengths primarily under 150 bp (**Figure 1F**). Moreover, the lengths of these novel lncRNAs were predominantly enriched in the 400-800 bp range (**Figure 1G**).

**Table 3:** Summary of reads mapping to the reference genome.

Sample name	Total Reads	Mapped Reads	Uniq Mapped Reads	Multiple Mapped Reads	Reads Map to’+’	Reads Map to’-’
Con1	98012958	92083053 (93.95%)	89304336 (91.11%)	2778717 (2.84%)	47899998 (48.87%)	47796079 (48.77%)
Con2	101561342	94874547 (93.42%)	91949415 (90.54%)	2925132 (2.88%)	49476905 (48.72%)	49258064 (48.50%)
Con3	104759978	100030662 (95.49%)	97270116 (92.85%)	2760546 (2.64%)	51841127 (49.49%)	51793587 (49.44%)
TB1	104881898	101151571 (96.44%)	98194841 (93.62%)	2956730 (2.82%)	52609213 (50.16%)	52471164 (50.03%)
TB2	109184910	104844268 (96.02%)	101898427 (93.33%)	2945841 (2.70%)	54414786 (49.84%)	54354169 (49.78%)
TB3	109461976	104090948 (95.09%)	101498790 (92.73%)	2592158 (2.37%)	53850676 (49.20%)	53748046 (49.10%)

To elucidate the involvement of lncRNAs in pre-metastatic pulmonary tissue, we conducted a comparative analysis of lncRNA expression between normal and pre-metastatic lungs. The findings revealed a pronounced divergence in lncRNA expression patterns between two groups, as depicted in the volcano plot and MA plot (**Figures 1H, I**). Utilizing the criteria with a fold change of ≥ 1.5 and a p-value of < 0.05, 232 significantly dysregulated lncRNAs were identified, comprising 101 upregulated and 131 downregulated lncRNAs (**Figure 1J**). These results revealed that there was obvious lncRNA expression difference between normal and pre-metastatic lungs.

### 3.2 Function prediction analyses of differentially expressed lncRNAs

**Figure 2:**
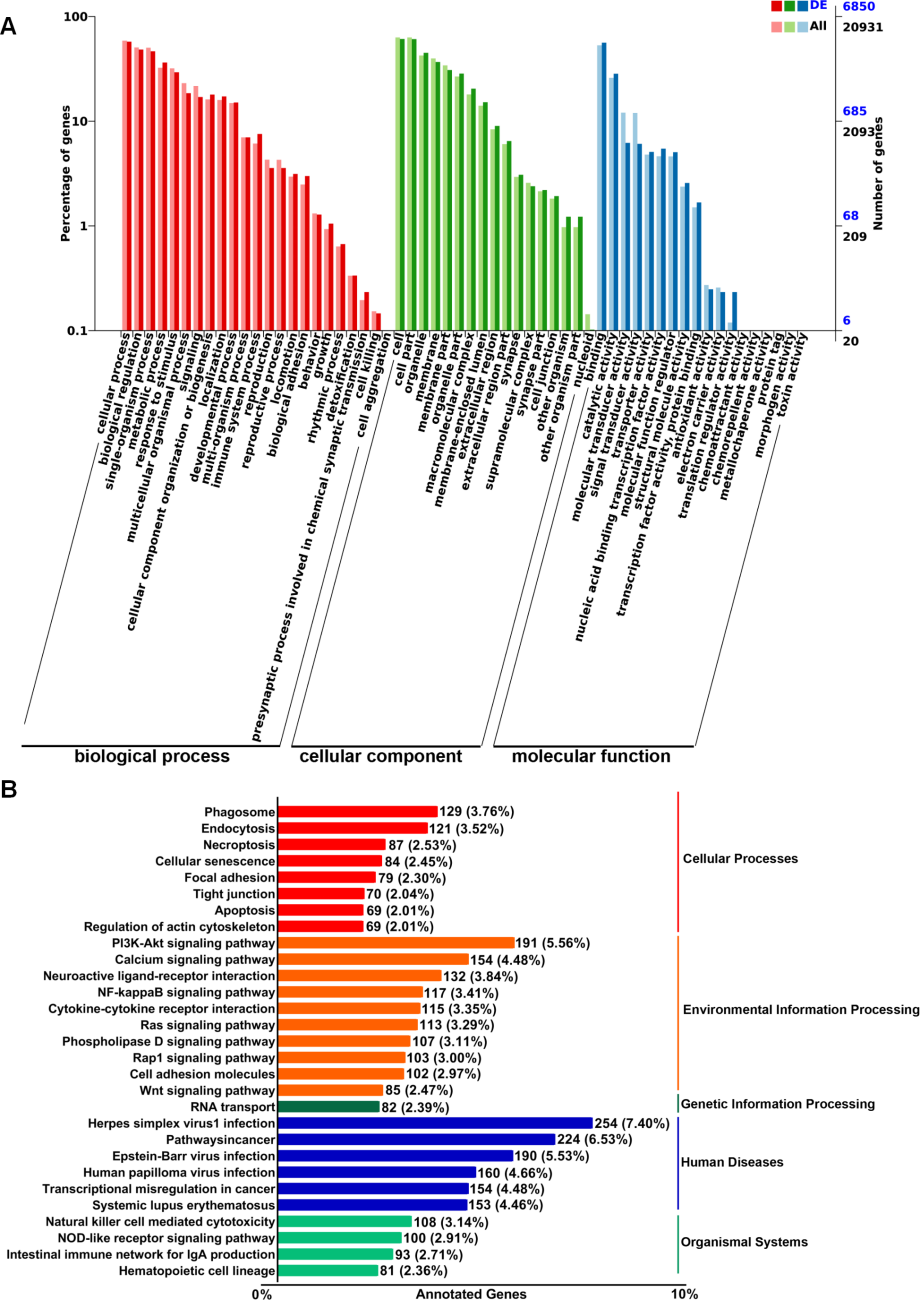
GO and KEGG pathway enrichment analysis of target genes regulated by differentially expressed lncRNAs in murine normal and pre-metastatic lungs. **(A)** GO annotation of differentially expressed lncRNA-targeting genes in terms of biological processes, cellular components, and molecular functions. **(B)** KEGG pathway analysis of differentially expressed lncRNA-targeting genes.

To explore the putative functionalities of the dysregulated lncRNAs in pre-metastatic lungs, Gene Ontology (GO) terms and Kyoto Encyclopedia of Genes and Genomes (KEGG) pathway analysis were performed on account of the target genes regulated by these lncRNAs in cis- and trans-acting manners. The most enriched GO items were cellular process, biological process, single-organism process, and metabolic process (**Figure 2A**). Moreover, binding, catalytic activity, molecular transducer activity, and signal transducer activity were the most prominent items in terms of molecular function (**Figure 2A**). KEGG pathway analysis indicated associations between the dysregulated lncRNAs and cellular processes such as phagosome formation, endocytosis, and necroptosis (**Figure 2B**). Furthermore, the top four environmental information processing pathways linked to these lncRNAs included the PI3K-Akt signaling pathway, calcium signaling pathway, neuroactive ligand-receptor interactions, and the NF-κB signaling pathway (**Figure 2B**). Correlation analysis further demonstrated a significant relationship between these lncRNAs and human diseases, particularly herpes simplex virus 1 infection and cancer pathways (**Figure 2B**). These results suggest that the dysregulated lncRNAs are associated with the modulation of multiple signaling pathways that may affect the establishment of the PMN.

### 3.3 Validation of differentially expressed lncRNAs via RT-qPCR

**Figure 3:**
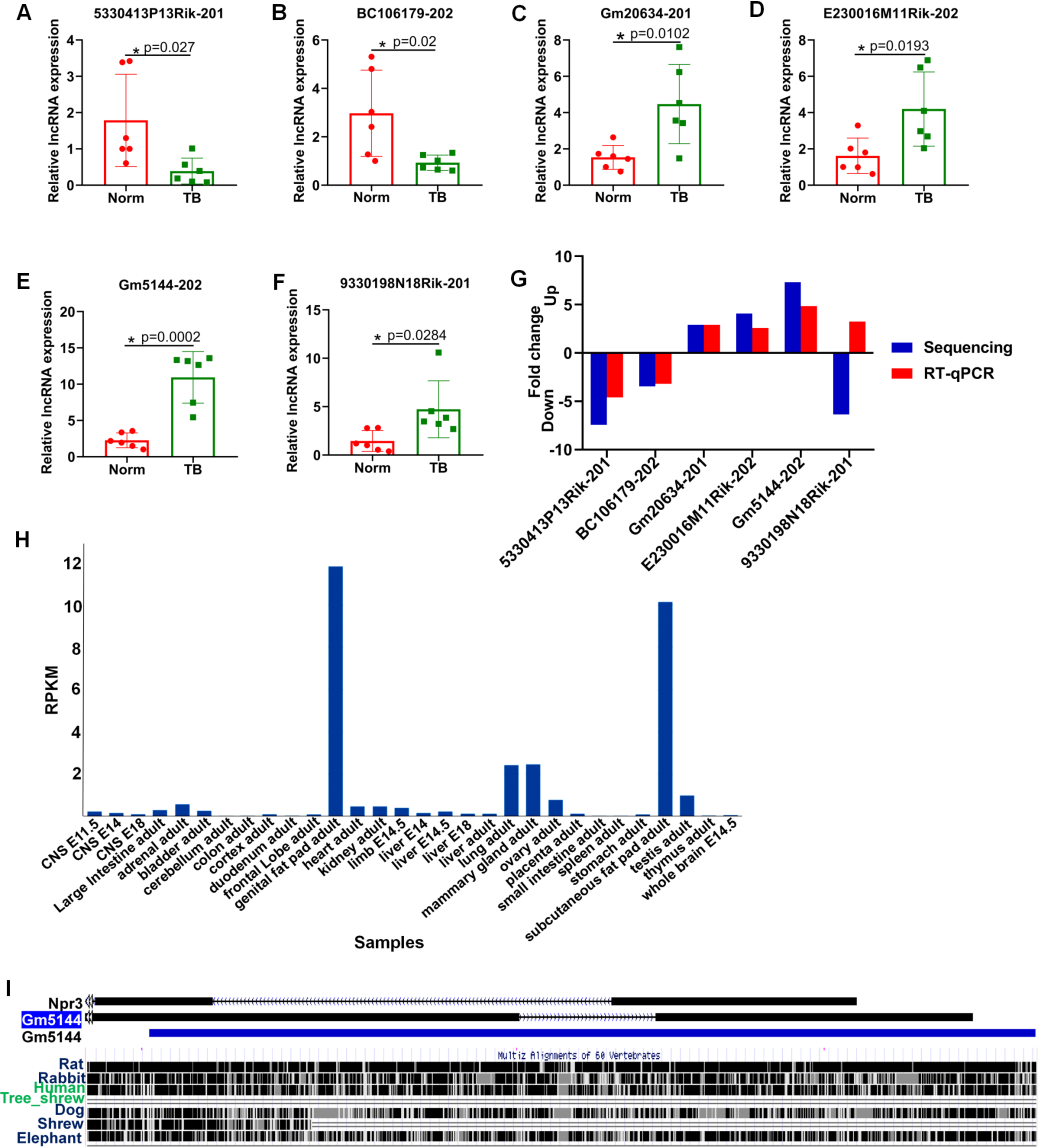
RT-qPCR validation of six differentially expressed lncRNAs in murine normal and pre-metastatic lungs. **(A–F)** Validation of ENSMUST00000140443 (lncRNA 5330413P13Rik-201) **(A)**, ENSMUST00000231570 (lncRNA BC106179-202) **(B)**, ENSMUST00000090779 (lncRNA Gm20634-201) **(C)**, ENSMUST00000147548 (lncRNA E230016M11Rik-202) **(D)**, ENSMUST00000227557 (lncRNA Gm5144-202) **(E)**, and ENSMUST00000146702 (lncRNA 9330198N18Rik-201) **(F)** expression in murine normal and pre-metastatic lungs via RT-qPCR. Norm denotes normal lungs, and TB denotes pre-metastatic lungs. **(G)** Comparison of fold change for the six selected lncRNAs. N=6, per group. *p < 0.05. **(H)** lncRNA Gm5144-202 expression pattern from NCBI database. **(I)** Conservative analysis of Gm5144-202 from UCSC Genome Browser.

To further confirm the differential expression of lncRNAs identified through high-throughput sequencing, we employed RT-qPCR analysis. Six lncRNAs, including three downregulated and three upregulated, were selected based on p-value, expression level, and consistent differential expression across groups. Consistent with the sequencing data, the expression levels of ENSMUST00000140443 (lncRNA 5330413P13Rik-201), ENSMUST00000231570 (lncRNA BC106179-202), ENSMUST00000090779 (lncRNA Gm20634-201), ENSMUST00000147548 (lncRNA E230016M11Rik-202), and ENSMUST00000227557 (lncRNA Gm5144-202) were significantly altered between normal and pre-metastatic lungs, with lncRNA Gm5144-202 exhibiting the most significant differential expression (**Figures 3A–E**). However, ENSMUST00000146702 (lncRNA 9330198N18Rik-201) was found to be upregulated in pre-metastatic lungs by RT-qPCR, which contradicted the sequencing data trend (**Figure 3F**). Based on the concordance of sequencing and RT-qPCR results, and the expressing difference, lncRNA Gm5144-202 was selected for further investigation (**Figure 3G**). Further bioinformatics analysis showed that lncRNA Gm5144-202 was highly expressed in the lungs besides in fat pads as per the NCBI database (https://www.ncbi.nlm.nih.gov/gene/) (**Figure 3H**), and it is highly conserved across mouse, rat, and human genomes according to the UCSC Genome Browser (http://genome.ucsc.edu/) (**Figure 3I**).

### 3.4 Upregulation of lncRNA Gm5144-202 in alveolar macrophages of pre-metastatic lungs

**Figure 4:**
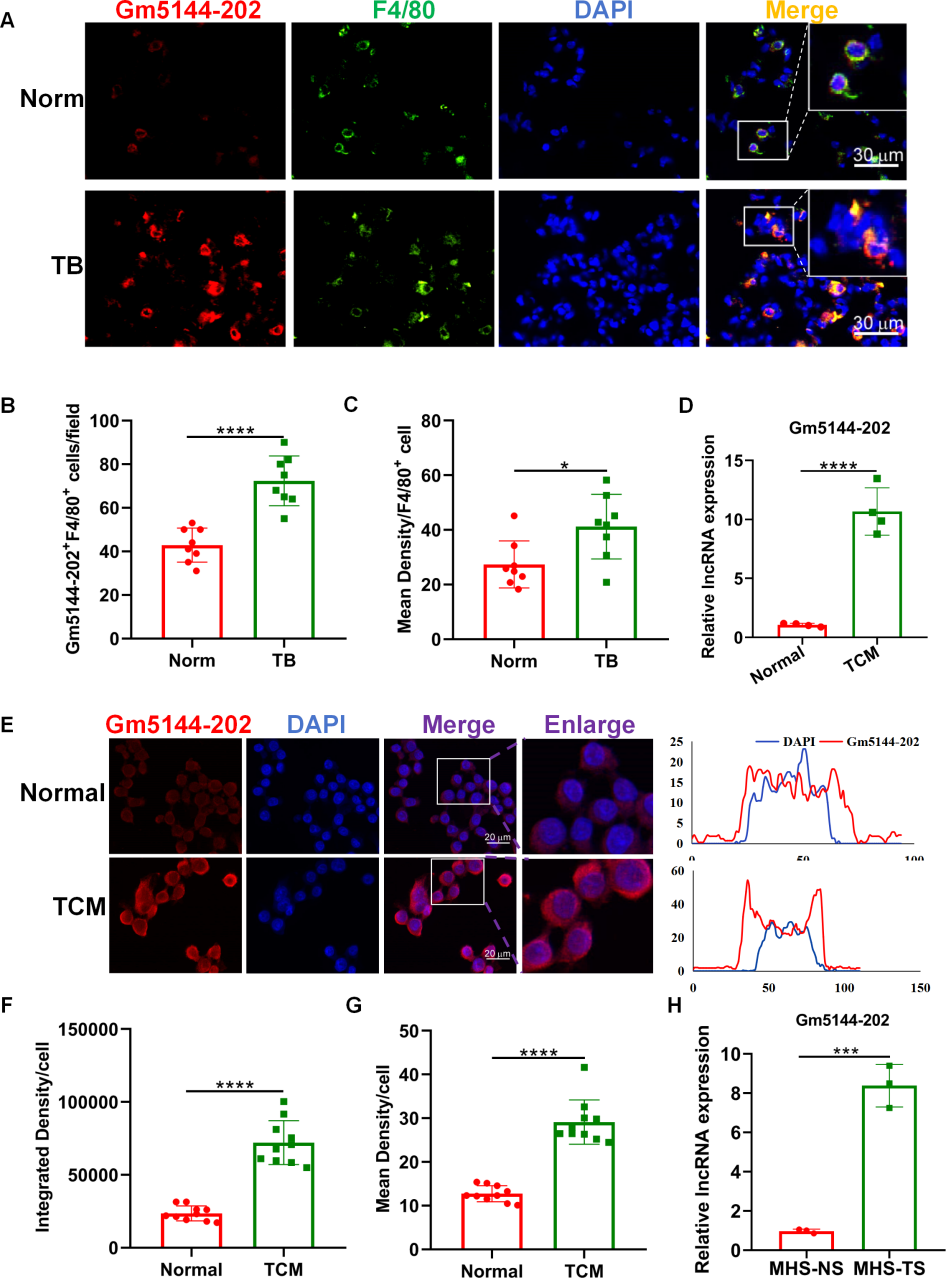
Upregulation of Gm5144-202 in alveolar macrophages (AMs) under tumor-bearing conditions. **(A)** FISH and F4/80 immunofluorescence staining in normal (Norm) and pre-metastatic (TB) murine lungs. Scale bar, 30 μm. **(B)** Quantitative analysis of Gm5144-202^+^F4/80^+^ cells per field (n=8). **(C)** Quantitative measurement of mean fluorescence intensity of Gm5144-202^+^ in F4/80^+^ cells (n=8). **(D)** RT-qPCR analysis of lncRNA Gm5144-202 expression in MH-S cells under normal medium (Normal) and B16F10-conditioned medium (TCM). **(E)** FISH analysis of lncRNA Gm5144-202 in MH-S cells under normal and TCM conditions (left), and fluorescence intensity of lncRNA Gm5144-202 and DAPI in a representative merged cell (right). Scale bar, 20 μm. **(F)** Quantitative assessment of integrated density per cell for Gm5144-202, n=10. **(G)** Quantitative evaluation of mean density per cell for Gm5144-202, n=10. **(H)** RT-qPCR quantification of lncRNA Gm5144-202 expression in MH-S cells exposed to normal mice serum (MHS-NS) and tumor-bearing mice serum (MHS-TS). *p < 0.05, ***p < 0.001, ****p < 0.0001, n.s, no significance.

Macrophages, as predominant immune cells in the pre-metastatic microenvironment, are pivotal in influencing tumor cell arrest, extravasation, and early colonization within the secondary tumor microenvironment, thus serving as potential therapeutic targets (21–23). Consequently, we assessed the expression of lncRNA Gm5144-202 in macrophages from both normal and pre-metastatic lungs. FISH analysis demonstrated significant localization of lncRNA Gm5144-202 within macrophages (**Figure 4A**). Additionally, lncRNA Gm5144-202 expression was markedly upregulated in pre-metastatic lungs compared to normal lungs (**Figure 4A**), corroborated by statistical analysis in **Figures 4B, C**. AMs represent the predominant macrophage subtype within the pulmonary system, and our previous findings have demonstrated that these cells accumulate in the pre-metastatic lung microenvironment, thereby facilitating lung metastasis in murine models of tumors (19, 24). Therefore, we then examined lncRNA Gm5144-202 expression in alveolar macrophage MH-S cell lines. RT-qPCR results revealed a substantial increase in lncRNA Gm5144-202 upon treatment with TCM (**Figure 4D**). Additionally, FISH analysis revealed that the expression levels of lncRNA Gm5144-202 were significantly increased in alveolar macrophages following TCM intervention, with this upregulation primarily localized to the cytoplasmic compartment (**Figures 4E–G**). Similar upregulation was observed in MH-S cells treated with serum from tumor-bearing mice (TS) (**Figure 4H**). Collectively, these findings indicate that lncRNA Gm5144-202 is upregulated in alveolar macrophages under tumor conditions.

### 3.5 LncRNA Gm5144-202 is associated with pro-tumor macrophage polarization and pre-metastatic niche formation

**Figure 5:**
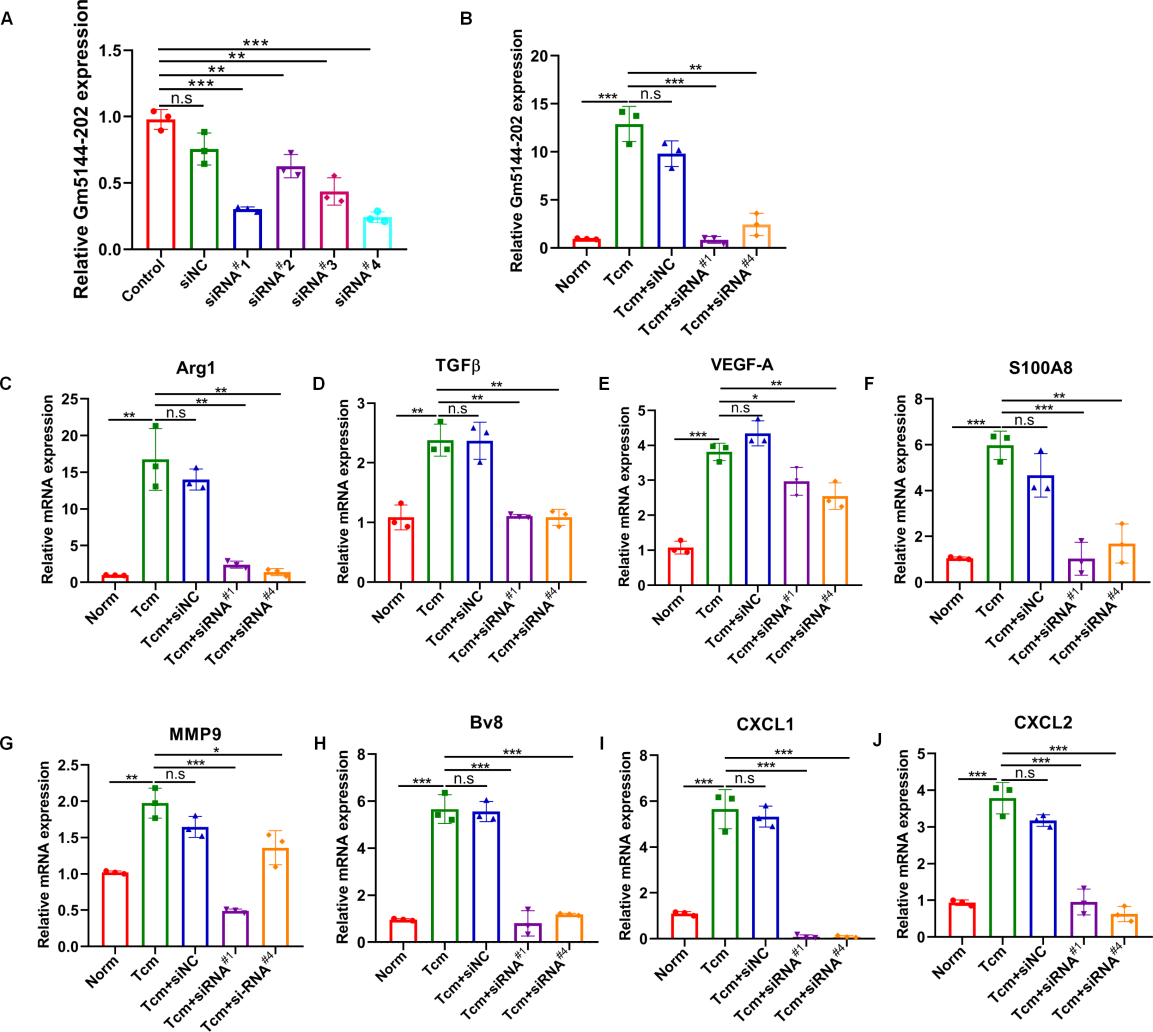
Silencing of Gm5144-202 in AMs impeded macrophage M2 polarization and PMN formation. **(A)** RT-qPCR analysis of Gm5144-202 expression following transfection of MH-S cells with siRNA negative control (siNC), siRNA Gm5144-202^#1^ (siRNA^#1^), siRNA Gm5144-202^#2^ (siRNA^#2^), siRNA Gm5144-202^#3^ (siRNA^#3^), and siRNA Gm5144-202^#4^ (siRNA^#4^) individually. **(B)** RT-qPCR quantification of Gm5144-202 expression in MH-S cells transfected with siNC, siRNA^#1^, or siRNA^#4^ in the condition of TCM or not. **(C–K)** RT-qPCR analysis of Arg1 **(C)**, TGF-β **(D)**, VEGF-A **(E)**, S100A8 **(F)**, MMP9 **(G)**, Bv8 **(H)**, CXCL1 **(I)**, and CXCL2 **(J)** expression in MH-S cells transfected with siNC, siRNA^#1^, or siRNA^#4^. The experiments were repeated at least three times. *p < 0.05, **p < 0.01, ***p < 0.001, n.s, no significance.

Previous studies have indicated that the tumor-bearing microenvironment can condition AMs into an M2 tumor-promoting phenotype, thereby facilitating distant lung metastasis (19, 25). To elucidate the role of lncRNA Gm5144-202 in M2 polarization, we quantified markers representative of the M2 phenotype through loss-of-function experiments. We initially transfected four siRNAs to evaluate knockdown efficiency in MH-S cells, with si-Gm5144-202^#1^ (siRNA^#1^) and si-Gm5144-202^#4^ (siRNA^#4^) being selected for further analysis (**Figure 5A**). Similar knockdown efficiencies were observed in MH-S cells with TCM treatment (**Figure 5B**). Notably, the expression levels of arginase-1 (Arg1), TGFβ, as well as VEGF-A were remarkably elevated in MH-S cells exposed to TCM compared to normal controls, but was markedly reduced following lncRNA Gm5144-202 silencing (**Figures 5C–E**). Additionally, cytokines and chemokines implicated in PMN formation, including S100A8, MMP9, Bv8, CXCL1, and CXCL2, were elevated in MH-S cells treated with TCM, while knockdown of lncRNA Gm5144-202 attenuated their upregulation (**Figures 5F–J**). These data collectively suggest that lncRNA Gm5144-202 in AMs modulates M2 macrophage polarization and contributes to pre-metastatic niche formation.

### 3.6 LncRNA Gm5144-202 in AMs modulates the motility of B16F10 melanoma cells

**Figure 6:**
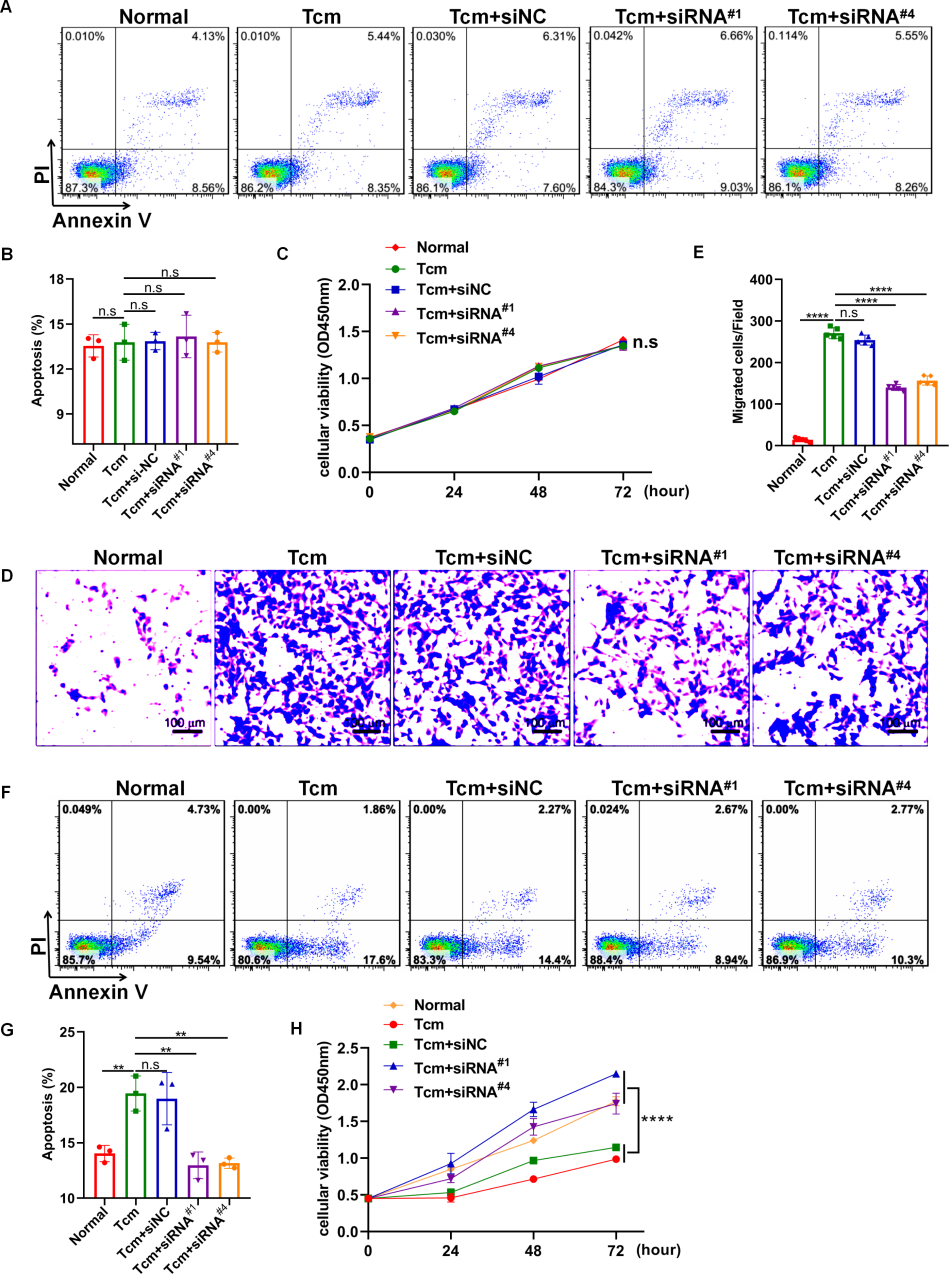
Downregulation of lncRNA Gm5144-202 in MH-S cells inhibited B16F10 cell migration. **(A)** Apoptosis analysis of B16F10 cells in a coculture system with 30% conditioned medium from MH-S cells transfected with siNC, siRNA^#1^, or siRNA^#4^. **(B)** Quantitative analysis of apoptotic cell percentages. **(C)** CCK8 assay depicting the proliferation of B16F10 cells in a coculture system with 30% conditioned medium from MH-S cells transfected with siNC, siRNA^#1^, or siRNA^#4^. **(D, E)** Transwell assay illustrating the migration of B16F10 cells in upper chambers with lower chambers containing MH-S cells transfected with siNC, siRNA^#1^, or siRNA^#4^. Scale bar, 100 μm. **(F)** The apoptosis of MH-S cells after transfected with siNC, siRNA^#1^, or siRNA^#4^. **(G)** Quantitative analysis of the percentages of apoptotic MH-S cells after transfected with siNC, siRNA^#1^, or siRNA^#4^. **(H)** The CCK8 assay showing the proliferation of MH-S cells after transfected with siNC, siRNA^#1^, or siRNA^#4^. The experiments were repeated at least three times. **p < 0.01, ***p < 0.001, ****p < 0.0001, n.s, no significance.

**Figure 7:**
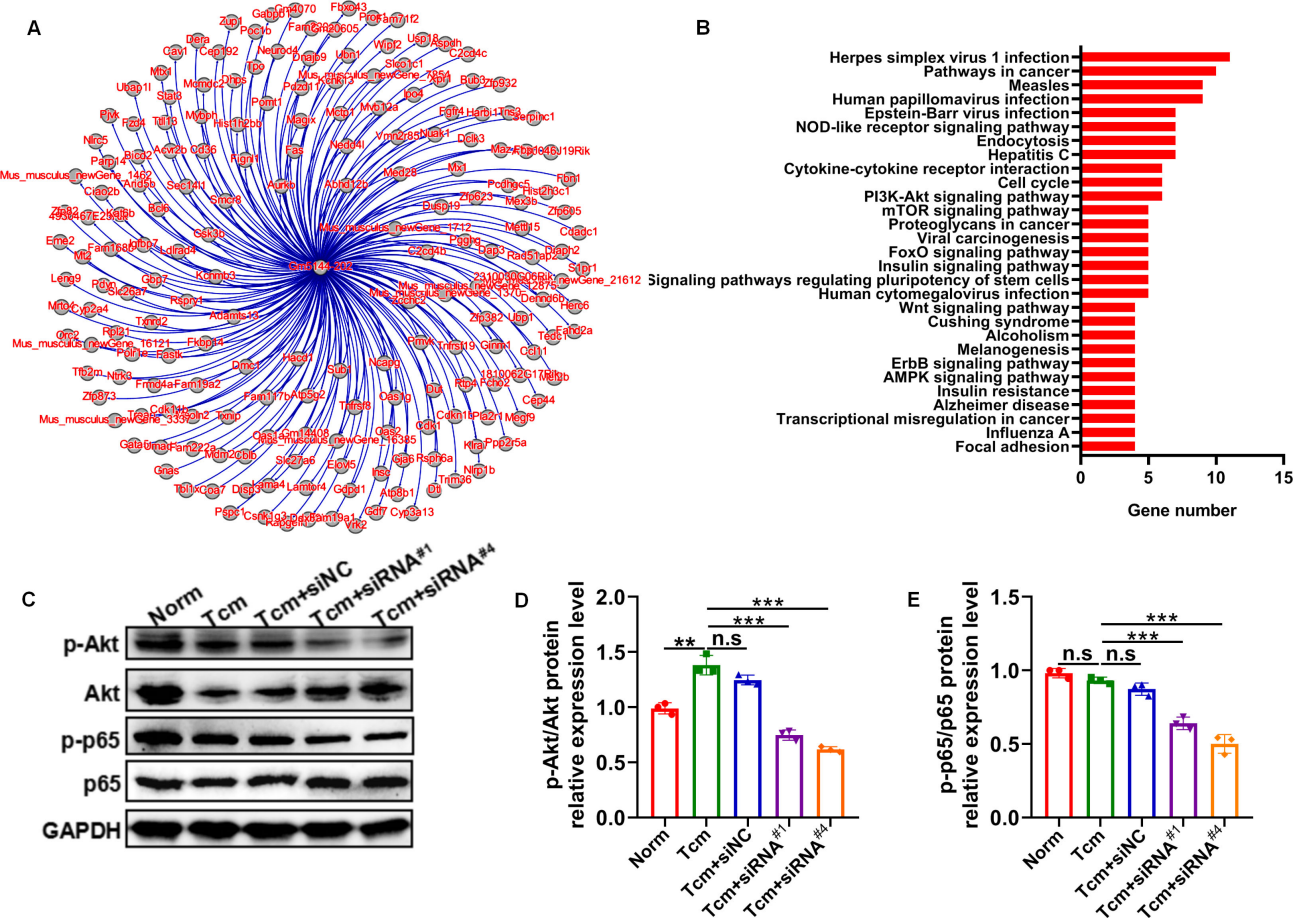
Predicted target genes and KEGG pathway analysis of lncRNA Gm5144-202. **(A)** Regulatory target gene network diagram for lncRNA Gm5144-202. **(B)** KEGG pathway analysis for lncRNA Gm5144-202. **(C)** Protein levels of phosphorylation- and total-Akt and NF-κB p65 in MH-S cells with siNC, siRNA^#1^, or siRNA^#4^ transfection detected by western blot. **(D, E)** Semiquantitative analysis of the phosphorylation- and total Akt and NF-κB p65 proteins. n = 3. **p < 0.01, ***p < 0.001, n.s, no significance.

It has been well-established that macrophage polarization facilitates the proliferation and metastasis of tumor cells (26, 27). Then, roles of lncRNA Gm5144-202 in regulating tumor cell proliferation, apoptosis, and migration were investigated. Initially, B16F10 cells were cocultured with 30% conditioned medium from MH-S cells that were with lncRNA Gm5144-202 knockdown. Observations indicated no significant alterations in apoptosis of B16F10 cells compared to the normal group (**Figures 6A, B**). Similarly, the proliferation rate of B16F10 cells remained unchanged as assessed by the CCK8 assay (**Figure 6C**). Notably, there was an obvious reduction in the migratory capacity of B16F10 cells under the influence of 30% conditioned medium from MH-S cells with lncRNA Gm5144-202 knockdown (**Figures 6D, E**). Nonetheless, the attenuation of lncRNA Gm5144-202 expression in MH-S cells markedly enhanced cellular proliferation, concurrently resulting in a reduction of apoptosis (**Figures 6F–H**). To clarify the molecular regulatory mechanisms of lncRNA Gm5144-202 in AMs, the cis- and trans-regulated target genes were predicted and listed. As shown in **Figure 7A**, 189 target genes were forecasted, with 2 target genes, including Sub1 and Mus_musculus_newGene_21612 were cis-regulated target genes, and the lasting were trans-regulated target genes (**Figure 7A**). Subsequent KEGG analysis of lncRNA Gm5144-202-regulated target genes highlighted significant enrichment in terms related to viral infection, oncogenic pathways, NOD-like receptor signaling, and the PI3K-Akt pathway (**Figure 7B**). Given that the PI3K-Akt/NF-κB signaling pathway has been implicated in the regulation of M2 macrophage polarization, and that lncRNA Gm5144-202 is predicted to participate in the PI3K-Akt pathway, we subsequently examined the molecular expression alterations within the PI3K-Akt/NF-κB signaling cascade. As anticipated, silencing of lncRNA Gm5144-202 markedly reduced the relative phosphorylation levels of Akt and NF-κB, indicating that lncRNA Gm5144-202 takes part in modulating the PI3K-Akt/NF-κB signaling pathway (**Figures 7C–E**). Collectively, these findings underscore the critical role of lncRNA Gm5144-202 in AMs in enhancing the migratory potential of tumor cells.

## 4 Discussion

The cellular constituents and molecular components in distant metastatic sites collectively dictate the destiny of metastatic cancer cells, providing not only a conducive microenvironment but also acting as dynamic facilitators for enhancing tumor proliferation, invasion, neovascularization, and immunosuppression. Elucidating the mechanisms underlying the preferential metastasis of disseminated tumor cells are emerging as a focal point in the research related to cancer metastasis (28, 29). During tumor progression, the PMN is subverted and reconstructed to assist the disseminated tumor cells for successful arrest and survival, culminating in cancer metastasis. Despite substantial advancements, the fundamental molecular mechanisms governing PMN formation remain inadequately understood and warrant further investigation. Beyond genetic alterations, epigenetic modifications have been shown to significantly contribute to PMN establishment. Accumulating evidence indicates that various miRNAs mediate PMN formation and distal metastasis (30–32), and recently, lncRNAs were also identified as one of the most essential epigenetic regulators for PMN formation. For instance, LINC00482 was found to competitively bind to miR-142-3p to regulate TGF-β1, thereby inducing microglial M2 polarization, influencing the pre-metastatic niche, and promoting brain metastasis in non-small cell lung cancer (NSCLC) (33). CSC-like CD90^+^ liver cancer cells released lncRNA H19, which modulated endothelial cells to promote angiogenesis and tumor cell adhesion to the endothelial cell monolayer (34). According to the “seeds and soil” theory, and considering that cancer cell-secreted exosomes can induce organotropic metastasis (35), numerous studies have emphasized the roles of lncRNAs packaged in extracellular vesicles released by tumor cells. However, not only do exosome-derived lncRNAs from tumor cells play critical roles, but those located within the PMN also significantly affect gene expression and cellular processes. In this study, we characterized, for the first time to our knowledge, the lncRNA expression pattern in pre-metastatic lungs and underscored the potential functions of differentially expressed lncRNAs. Notably, the dysregulated lncRNAs were deduced to be highly associated with cellular processes such as phagosome formation, endocytosis, and necroptosis, and were linked to the PI3K-Akt signaling pathway, calcium signaling pathway, neuroactive ligand-receptor interactions, and the NF-κB signaling pathway.

There is substantial documentation indicating that the conversion of macrophages to immunosuppressive phenotypes is a fundamental requirement for PMN formation. The alternative activation of pro-tumor macrophage phenotypes is associated with various malignancy processes, including cancer cell proliferation, invasion, matrix remodeling, angiogenesis, and suppression of adaptive immunity (36). Cytokines such as IL-4 and IL-13 within the tumor microenvironment regulate the polarization of tumor-associated macrophages (TAMs) through downstream Stat6-dependent inhibition of Arg1, activation of PPARγ, and TSC1-mediated inhibition of mTOR. Additionally, tumor-derived exosomes have been validated to polarize macrophages toward an immunosuppressive phenotype via glycolytic metabolism reprogramming (21). Simultaneously, evidence suggests that lncRNAs are involved in modulating immune cell differentiation and function, with some lncRNAs specifically modulating macrophage polarization. For instance, silencing lncRNA NR_109 in M2 macrophages significantly hindered IL-4 induced M2 macrophage polarization and depressed their capacity to support tumor cell proliferation and metastasis (37). Knockdown of lncRNA-MM2P inhibited cytokine-induced M2 macrophage polarization and impaired macrophage-mediated tumorigenesis, tumor growth *in vivo*, and tumor angiogenesis (38). Colorectal cancer (CRC) cell exosome-derived lncRNA RPPH1 mediates macrophage M2 polarization, promoting CRC cell metastasis and proliferation (39). Silencing SNHG1 inhibited M2 macrophage polarization by suppressing STAT6 phosphorylation, thereby inhibiting tumor growth and angiogenesis (40). In our investigation, we initially employed RNA-seq technology to identify differentially expressed lncRNAs in pre-metastatic lung tissues, pinpointing a novel lncRNA, Gm5144-202, which exhibited elevated expression levels in M2-like macrophages. Subsequent knockdown of lncRNA Gm5144-202 in MH-S cells hindered TCM-induced M2 polarization and delayed the establishment of the pre-metastatic niche. Collectively, these findings highlight the potential clinical significance of lncRNA Gm5144-202 in mitigating lung metastasis of cancer.

To elucidate the mechanistic role of lncRNA Gm5144-202 in M2 polarization and PMN construction, we conducted target gene identification and KEGG pathway analysis. This investigation revealed that the target genes of lncRNA Gm5144-202 are implicated in various biological pathways, including herpes simplex virus 1 infection, oncogenic signaling, and human papillomavirus infection, indicating a significant correlation between lncRNA Gm5144-202, immune responses, and oncogenesis. Additionally, protein expression analyses demonstrated that the activity of lncRNA Gm5144-202 was mediated via the PI3K/Akt-NFκB signaling cascade. Numerous studies have demonstrated that suppressive and regulatory immune cells are crucial for creating a microenvironment conducive to tumor cell engraftment and colonization. Our study further demonstrated that lncRNA Gm5144-202 significantly promotes tumor cell migration and M2 polarization, suggesting its potential role in clinical prevention and treatment of tumor metastasis. Emerging evidence underscores the pivotal function of long non-coding RNAs (lncRNAs) in the diagnosis and prevention of tumors. For instance, high expression of lncRNA LINC00926 predicted a good clinical outcome of breast cancer (41). LINC00115 was identified as a novel regulator of chemotherapy-resistant breast cancer stem-like cells and high LINC00115 expression is linked to a poor prognosis in breast cancer patients post-chemotherapy (42). These findings suggest a promising future for lncRNA in tumor prediction and treatment.

Despite significant progress, there are still limitations to our study. First, besides lncRNA Gm5144-202, the remaining 4 lncRNAs that exhibited significant difference needs further investigation to explore their roles in regulating lung metastasis. Second, the specific regulatory mechanisms of lncRNA Gm5144-202 remain to be further elucidated and require validation through function experiments. Additionally, preclinical animal studies are imperative to confirm the *in vivo* therapeutic efficacy, thereby substantiating lncRNA Gm5144-202 as a viable therapeutic target. It is also crucial to verify the clinical relevance of lncRNA Gm5144-202 in predicting and preventing lung metastasis.

In summary, our study is pioneering in exploring the expression profile of lncRNAs and the potential functions of differentially expressed lncRNAs in pre-metastatic lungs, providing a framework for screening functional lncRNAs in PMN. Moreover, lncRNA Gm5144-202, significantly upregulated in AMs of pre-metastatic lungs, has been verified to play a role in M2 polarization and PMN formation, underscoring its potential in reducing the occurrence and recurrence of lung metastasis.

## Data Availability

The datasets presented in this study can be found in online repositories. The names of the repository/repositories and accession number(s) can be found below: https://www.ncbi.nlm.nih.gov/,GSE274656.
